# The Dilemma and Path of Rural Environmental Governance in China: From the Perspective of a Community with a Shared Future

**DOI:** 10.3390/ijerph20021446

**Published:** 2023-01-13

**Authors:** Yigang Zhang, Xiaoyan Guo

**Affiliations:** School of Law, Qufu Normal University, Rizhao 276800, China

**Keywords:** rural environmental governance, community with a shared future, cooperative governance, environmental legislation

## Abstract

With the aim of achieving the goal of ecological livability in Chinese rural society, the issue of rural environmental governance has received great attention from the CCP and the government. However, due to local governments’ model of development in exchange for economic interests and the “urban and rural binary” structure of environmental governance, rural environmental governance faces many dilemmas, such as lack of normative standards, lack of environmental governance subjects, and lack of judicial security. In order to improve the development of rural ecological civilization and realize ecologically friendly agriculture, this paper proposes a solution path for rural environmental governance from the perspective of the idea of the community with a shared future for humanity. Specifically, this solution path includes establishing the concepts of cooperation and governance of environmental protection, improving villagers’ participation in environmental protection, balancing economic and environmental interests in rural environmental governance, and building a long-term mechanism for the rule of law in rural environmental governance.

## 1. Introduction

In the 19th National Congress of the Communist Party of China in 2017, the party proposed the implementation of the rural revitalization strategy with the aim of realizing eco-friendly and livable governance [1]. In March 2020, the General Office of the CPC Central Committee issued its Guiding Opinions on Building a Modern Environmental Governance System, aiming to establish “an environmental governance system with clear guidance, science-based decisions, rigorous enforcement, effective stimulation, diversified participants, and sound interactions” by 2025. This series of statements highlighted the development direction for rural ecological and environmental governance in the new era. Governance concerns for rural areas have taken a qualitative leap, from the benchmark material level of solving the problems of food, clothing, compulsory education, basic medical services, and safe housing to the new height of healthy and green rural production and life. In accordance with the overall requirements for thriving businesses, pleasant living environments, social etiquette and civility, effective governance, and prosperity in rural areas, China aims to implement a blueprint for beautiful countryside and rural revitalization. In short, everyone is looking forward to enjoying green, low-carbon, ecologically livable, and healthy lives with high living quality. However, through on-the-spot investigation of China’s rural environmental sampling formula, it can be observed that rural environmental pollution is serious and the need for rural environmental governance is extremely urgent. Regarding this topic, current academic circles have pursued the following research paths. First of all, macroscopic analysis of the founding of the new China over the past 70 years has shown that rural environmental governance has gone through five stages, and the logic of transformation has been analyzed [2]. Second, special-subject studies and interpretations have been conducted for particular aspects of rural environment, such as the sanitation environment [3] sewage treatment [4], and the living environment [5]. Third, different theoretical models have been used to analyze rural environmental governance issues, such as [6], who employed the perspective of planned behavior theory to analyze the dilemma in the model of direct government-controlled governance of the rural ecological environment at the present stage [7], who proposed a path to achieve a positive interaction between government-led and farmer participation in rural environmental governance based on the “embeddedness theory” of action; and [8] who analyzed the “idle” phenomenon in rural environmental policy based on a fuzzy-conflict framework. As a fourth stage, based on the analysis of the current situation of rural environmental pollution and its governance, five relevant dimensions of climate governance were analyzed through a systematic literature review of climate governance research [9]. This paper analyzes the dilemma of rural environmental governance at the level of rule of law and proposes multiple paths to improve rural environmental governance based on the perspective of the community with a shared future for humanity.

1. The current situation of China’s rural environmental pollution and its governance

In a general sense, rural environmental governance refers to the activity of political actors in managing the operation of the rural environment in an integrated manner with the help of certain concepts, rules, institutions, resources, mechanisms, etc. [10]. The realization of environmental rights is associated with a series of terms, such as “clean, healthy, good, ecologically balanced, sustainable, free from pollution, and suitable for personal development” [11]. Environmental rights are closely related to the right to health of farmers. The right to health is firmly established in both domestic and international law and is often defined as “a state of complete physical, mental and social well-being”. The enjoyment of the highest standard of health is one of the fundamental rights of every human being. The Committee on Economic, Social and Cultural Rights (CESCR) notes that “the right to health encompasses a wide range of socio-economic factors that promote the conditions under which people lead healthy lives, and extends to the underlying determinants of health, such as food and nutrition, housing, access to safe drinking water and adequate sanitation, safe and healthy working conditions, and a healthy environment” [12]. 

In China, although the issue of rural environmental governance has received a great deal of attention at the national level, environmental problems in the vast rural areas are still serious due to factors such as the “urban centrism” caused by the long-standing dichotomy between urban and rural ecological environments. Rural environmental pollution can be summarized in four major aspects. First of all, water bodies suffer from pollution. Rural garbage disposal is not timely, production waste cannot be recycled by itself, and the phenomenon of randomly discharging garbage into rivers is serious, causing damage to the surface water environment. Excessive use of pesticides, soil films, fertilizers, etc., in agricultural production has resulted in long-term pollution of drinking water. As shown by statistics, non-point source pollution from China’s rural planting industry has resulted in total groundwater pollution rates of up to 81% for nitrogen and 93% for phosphorus [13], and the cancer death rate due to environmental pollution is much higher in rural society than in urban areas [14]. Second, there is the problem of domestic waste pollution. With the improvement in farmers’ living standards, waste output has increased, but, due to limited resources, waste disposal is not timely and the problem of piles of garbage remains prominent. Third, land pollution is serious. In order to increase the yield of agricultural products, farmers unscientifically use chemical fertilizers and pesticides in large quantities in the process of growing crops, causing the residual liquid and residues of agricultural chemicals to accumulate in the soil, resulting in obvious soil acidification, soil hardening, etc. Fourth, there is the double threat of urban pollution and industrial pollution transfer: industrial waste caused by illegal emissions from village enterprises, environmental pollution caused by rough tourism in rural areas, and secondary pollution brought about by the transfer of urban heavily pollutive and energy-intensive industries to rural areas. This section introduces new information and data as recommended by experts. Environmental pollution seriously damages the physical and mental health of human beings and infringes upon the right to life and health of farmers. It has been found that farmers may be exposed to multiple hazards, simultaneously or sequentially, through different exposure pathways and from different sources at different time periods, such as harmful compounds in the form of liquids, gases, dust, smoke, etc., and physical hazards in the form of noise, vibrations, temperature, radiation, etc. These can all damage farmers’ physical and mental health to varying degrees. About 170,000 farmers die each year worldwide as a result of their occupational activities, and millions suffer from occupational health problems [15]. In China, overuse of fertilizers and pesticides and the toxic residues of pesticides left in water pose great health risks to families in rural areas [16]. Studies have found that exposure to pesticides has a wide range of long-term and short-term effects on the health of farmers. The former involve the peripheral nervous system, white blood cells, liver, and electrolytes, while the latter involve blood cells, the liver, kidneys, electrolytes, and the peripheral nervous system [17]. 

However, the damage caused by environmental pollution is long-term and hidden, and the relationship between damage actions and damage results is complex. On China Judgements Online, using “environmental pollution damage compensation” as the key words shows that there are almost no cases where farmers as the main body have requested environmental pollution damage compensation. Secondary pollution is brought about by the transfer of urban heavily pollutive and energy-intensive industries to rural areas. Furthermore, the current rural environmental governance system in China has the characteristics of being closed, exclusive, and subject to significant lag [18]. And there are governance problems such as “power suspension”, “identity dissolution”, “system idling” and “factor mismatch” in environmental governance [19]. In this context, paying attention to rural environmental issues, exploring effective ways to manage rural environment, and constructing a long-term mechanism for rural environmental governance are not only related to the living environment of thousands of farmers, but also related to the modernization of agricultural development, and the revitalization and sustainable development of rural society. 

## 2. Causes for the Lag in Rural Environmental Governance

(1) Governance ideas relating to urban centralism

There are fundamental reasons for the lag in China’s rural environmental governance, as well as various historical factors that have increased the complexity of China’s environmental governance in rural areas. Mao Zedong proposed the Four Modernizations as a strategic objective at the beginning of the establishment of the new China: modernization of agriculture, industry, national defense, and science and technology. However, the implementation of the Four Modernizations was not synchronized. Industrial modernization was addressed first and given priority in the development model, while modernization of agriculture, national security, and science and technology has been rather gradual. Under the pressure of gross domestic product (GDP) evaluation, local governments sacrificed environmental protection for economic development, which heightened the tensions between environmental and economic benefits. The urban–rural dual model is inevitable under the strategic orientation that gave priority to the development of heavy industry in Chinese history. This model reflects the serious imbalance in resource allocation between urban and rural environmental governance. In the context of rapid economic development, urban environmental governance issues have gradually started to receive attention. However, in a considerable part of rural society, solving poverty problems and increasing farmers’ incomes are still the first areas of concern for the government. The environmental interests of rural areas and farmers have not received due attention from the law, and the tendency toward “urban centralism” in environmental legislation is obvious [20]. Series of national and local laws and regulations, whether on pollutant emissions, environmental quality standards, supervision and management of the treatment of environmental pollution, environmental noise, or environmental health, show that the majority have an urban and industrial focus. With regard to rural areas, agriculture, and farmers, little legislation has been introduced to protect their rights, and most has involved principle-based clauses that lack operability. The state has concentrated its resources on fixing urban environmental problems and promoting the development of urban environmental governance, which has a certain historical rationale but has also led to insufficient attention being given to rural environmental problems [21].

(2) Lack of sustainable development perspective among grassroots governors

In addition to the neglect of the rural environment caused by the urban-centric environmental governance model, some local levels of government also lack an understanding of and research on the actual situation of the rural environment, blindly believing that rural areas still possess clean waters and green mountains. Moreover, other local levels of government lack a sustainable development view of rural environmental management and do not adequately recognize its importance. Furthermore, environmental governance in China is both vertical and horizontal; that is, it has a strip structure. Local environmental protection departments, as the executive bodies of local environmental regulation, are under the dual leadership of the block and the strip. When local economic development pressure is high, financial power and personnel power are limited by local governments’ environmental protection departments. Their environmental protection enforcement actions are subject to many restrictions, and they often act at the expense of the environment and implement lenient policies to attract investment [22]. Finally, rural environmental management also suffers from a shortage of funds and insufficient investment. According to the data published by the official website of the Ministry of Ecology and Environment, the central government arranged CNY 25.8 billion of special funds to support 150,000 administrative villages carry out environmental improvements during the 13th Five-Year Plan period. Approximately two-thirds of the administrative villages have not yet met the environmental improvement requirements, and the effectiveness of the improvements in the areas that have carried out such work is still unstable. Approximately three-quarters of the administrative villages have not completed sewage treatment improvements, the resource utilization level is not high, there is a serious lack of capital investment, the long-term mechanism is not perfect, and the effectiveness of governance is not obvious. It has been demonstrated that the shortage of funds for environmental management is exacerbated when they are distributed to local levels of government [23]. In other words, the lack of funds is a key constraint on environmental governance in rural areas [24]. The nature of the environment as a public good, strong externalities, the property rights of the commons, and the tragedy of the commons [25] all dictate that environmental governance cannot rely on the market alone [26], and that the government should assume a leading role in environmental governance [27].

## 3. The Dilemma of Legislation on Rural Environmental Governance

### 3.1. Inadequate Legislation on Rural Environmental Protection

Environmental legislation ensures the long-term development of environmental governance, but it is paradoxical that, in a society governed by the rule of law, there are so few rural-specific rules and regulations. On the one hand, as environmental pollution hazards have increased over the years, public awareness of environmental protection has grown, and the notion that “green maintains are themselves gold mountains” has become deeply ingrained in people’s hearts. At the same time, China has developed a rather well-established environmental legal system at the legislative level. At present, there are 33 environmental protection laws, 48 administrative regulations, and 94 departmental regulations pertaining to environmental protection.

On the other hand, since the existing laws and regulations primarily target urban areas, legislation on rural environmental protection is severely deficient. A conflict exists between unbalanced and inadequate legislative development on the one side and the ever-growing need for legislation in rural areas on the other. Up to now, China has not enacted legislation specifically targeting rural environmental protection, and the relevant regulations are scattered across various chapters and articles in the Environmental Protection Law of the People’s Republic of China, the Agriculture Law of the People’s Republic of China, the Water Law of the People’s Republic of China, the Animal Husbandry Law of the People’s Republic of China, the Law of the People’s Republic of China on Prevention and Control of Environmental Pollution by Solid Waste, the Law of the People’s Republic of China on Water and Soil Conservation, and the Land Administration Law of the People’s Republic of China. In terms of content, these laws and regulations focus on the general sense of environmental protection or on one particular aspect, such as water resources, land resources, and other special matters of protection. The overall lack of rural environmental protection must be targeted. These provisions regarding the protection of the environment in rural areas are dispersed and fragmented, and most of them are subordinate clauses in the legislation. There is potential for the requirements of various laws and regulations to come into direct conflict with one another. This is partly because farmers are rarely involved in environmental legislation and are at the bottom of the public participation gradient [28]. In addition, the provisions for the protection of the rural environment are highly principled and theoretically developed but difficult to put into practice. “Chinese environmental laws are more likely to contain very broad and literary-like language, often using vague language like ‘should’ and ‘encourage’ rather than mandatory language as ‘must’” [29]. As a result, they cannot directly guide environmental governance in rural areas. Last but not least, as rural environmental protection is a broad area, the shortcomings of unsystematic legislation and underdeveloped systematization have become apparent. Prevention and control of soil pollution, non-point source (NPS) pollution, and pollution from large-scale breeding of livestock and poultry, as well as control of environmental pollution transfer from urban to rural areas and ecological compensation for rural areas, have not yet been addressed in existing legislation on the rural environment.

### 3.2. Government-Centered Environmental Governance in Rural Areas

In rural areas, environmental governance centered on the government has been implemented for a long time and a top-down approach has been followed [30], which has led to a lack of overall planning for rural environment governance. The mechanism of pressure-based performance assessment serves as the driving force behind pursuit of short-term gains that does not take into consideration long-term effects. Some local governments respond slowly or even turn a blind eye to the deterioration of the rural environment. Under the pressure of inspection and task indicators from higher-level authorities, grassroots governments employ campaign-style governance, which involves various administrative agencies adopting centralized allocation of human, physical, and financial resources to accomplish goals in a short period of time. The campaign-style governance approach results in rapid effects but is hardly sustainable. Matland points out that local governments are prone to symbolic implementation when there is a high degree of ambiguity and conflict in policies [31]. In addition, “fragmentation” of local governments’ environmental behavior can be observed, and studies have shown that polluting firms evade environmental regulations by relocating. Reductions in regional pollution may even lead to an increase in national pollution, which prevents environmental regulation from having a scale effect and reduces the efficiency of environmental management [32]. As a result, farmers, whose demands are neglected, are relegated to the status of onlookers and bystanders, and their participation in the governance process is severely limited. Once inspection by superiors has been completed, environmental protection in rural areas is plunged into a situation where implementation is easier said than done [33]. Studies have shown that polycentric governance systems that include many institutions have higher quality environmental outputs than single-center government governance systems [34]. Collaborative governance appears to be more effective in creating, implementing, and complying with environmental systems [35]. On the other hand, as rural environmental management is a long-term systemic project, it necessitates the employment of specialized personnel, the application of cutting-edge technology, and the completion of infrastructural facilities; all of these aspects require financial backing. However, the financial investment that the state provides for rural environmental governance is on the lower end of the spectrum. Environmental governance sometimes adopts a campaign style when it is implemented by grassroots governments. Additionally, the vast majority of local governments take action in response to inspections rather than working to fundamentally resolve environmental issues.

Environmental organizations do not have adequate motivation to participate in rural environmental governance and, as a result, their practices are absent. The organizing institutes of the majority of environmental organizations are often set up in cities. These organizations concentrate mostly on environmental protection in well-known scenic areas, paying relatively little attention to the environment in rural areas. Environmental organizations lack the internal motivation to focus on rural environmental issues, and there are few examples of environmental organizations being concerned about rural environments in practice. Farmers, as the main stakeholders, should have been the core strength of environmental protection and governance in rural areas [36]. However, owing to ineffective primary-level governance and economic development levels in rural areas, farmers are more concerned with economic interests than environmental interests. Primary-level organizations in rural areas are weak in terms of governance, and farmers are constrained by the need to survive and develop and are more concerned with economic interests than environmental protection and environmental interests; thus, rural environmental protection can be easily trapped in the “collective action problem”. Olson (1965) defined collective action as any act of supplying a collective good, and the exclusivity of consumption is the main criterion for distinguishing collective goods from private goods [37]. Collective goods are non-exclusive or public; that is, the results of the action do not belong to the actor alone but are shared by the members of the collective [38]. As public goods, environmental benefits can be enjoyed collective members whether they take action or not, which causes the “free-rider” phenomenon. Collective members receive benefits at no cost and the activity of an individual economic actor can damage others without any cost to the person causing the external diseconomies. Social role theory suggests that there are individual differences in the behavior of actors with different social roles [39]; thus, heterogeneous individuals have different levels of willingness to participate in public environmental governance [40]. With regard to whether farmers will be willing to participate in public environmental governance, it has been suggested that the lower the perceived harm of environmental pollution, the more likely it is that “free-rider” behavior will occur [41]. The solution to the “free-rider” phenomenon mainly relies on an incentive mechanism; however, the effectiveness of traditional informal incentives, such as customs and public opinion, has been greatly diminished in the process of urbanization and modernization in rural societies.

### 3.3. Lack of Judicial Guarantees for Environmental Protection in Rural Areas

From the perspective of subjectivity, the ranks of judicial personnel in rural areas have poor awareness of environmental protection and lack professionalism. Judicial officials have not fully examined the importance of rural environmental governance for rural revitalization and ecological livability. Therefore, despite being the main body responsible for environmental public interest litigation, the procuratorate in rural areas rarely takes the initiative to participate in environmental governance. On the other hand, the concealment and long time-scale of ecological damage and environmental pollution lead to extensive environment-related cases requiring professional attention, which makes it difficult to obtain evidence and determine causal relationships in environmental litigation. At present, China lacks professional judicial officials in rural areas, and such areas do not meet the requirements for professional ranks of judicial personnel in the field of the environment and resources [21]. Moreover, in terms of the judicial environment, access to judicial procedures is difficult for two reasons. Firstly, most rural environmental cases are complex. Secondly, the cost of litigation is too high for farmers. Though farmers are victims of environmental pollution, both the poor predictability of verdicts and unfamiliarity with judicial procedures discourage them from appealing to the law. Farmers have difficulties in estimating reliable probabilities of winning lawsuits. A few may seek administrative assistance when their rights are violated, but the majority choose to remain silent and put up with environmental pollution.

## 4. Multiple Paths to Rural Environmental Governance Improvement

Since the 19th National Congress of the CPC, ecological development in rural areas and the implementation of eco-agriculture have been the key tasks outlined in the annual “No. 1 central document”. Agricultural ecological governance, agricultural ecological restoration, and the enhancement of the rural living environment have emerged as new fields of agricultural governance. The new direction of laws and regulations and the new changes in the “Three Rural” issues (issues relating to agriculture, rural areas, and farmers) indicate a shift from the urban–rural dual structure to urban–rural integration: the integrated development of urban and rural areas. The ecological environment relates to people’s livelihoods and rights and the rule of government by the people. Therefore, the old notion that rural agriculture must be sacrificed for urban industrial development must be abandoned and a new path for rural environmental governance from the perspective of the community with a shared future should be explored.

### 4.1. Establishing Cooperative Governance in Environmental Protection

Cooperative governance relies on multiple actors successfully working together to achieve a common goal on the basis of voluntariness and equality. Within the co-governance model, multiple actors are responsible for making crucial decisions and capable of empowering others to participate in actions equally. In practice, cooperative governance is based on emotional connections among individuals in an acquaintance society or contractual cooperation in an industrial society [42]. In terms of the environmental governance problems in rural areas, it is essential to change the predominant governance model, which is government-centered, and to uphold governance by multiple stakeholders. The principles of wide consultation, joint contribution, and shared benefits should be adhered to and the participation of multiple stakeholders advocated for in order to create a governance environment where everyone is responsible, everyone contributes, and everyone enjoys the benefits. In this way, the participation of multiple governance actors in building a modern rural environmental governance system can be promoted and the outcomes of rural environmental governance and green development shared [43]. The establishment of the concept of cooperative governance for environmental protection is in line with the thinking pertaining to the community with a shared future for humanity and its needs. The relevance, integrity, and externality of environmental resources mean that the government, village committees, and farmers are all stakeholders in environmental interests. A stakeholder refers to any individual or group that can affect the realization of an organization’s goals or that is affected by the process of an organization achieving its goals [44]. 

The key to establishing a community of shared environmental interests is raising community members’ environmental awareness. Farmers are stakeholders in the rural environment, and rural environment governance, which aims to ensure that farmers can enjoy the green water and blue sky, is conducive to physical and mental health. If the rural environment continues to deteriorate, it will also directly threaten the living environment of farmers. Therefore, farmers should change the traditional concept according to which “environmental management is the responsibility of the government” and establish a new concept according to which “ecological environment is everyone’s responsibility”, combining government-led initiatives and farmers’ autonomy. In the process of mobilizing farmers to participate in rural environmental governance, there is, in addition to the promotion of environmental protection concepts, a need to comprehensively examine various factors, such as farmers’ motivation to participate, risk tolerance, and level of awareness of environmental conditions and the impact of legal norms on farmers [45]. In short, farmers should be included in the environmental community of interest. Since the sources of rural pollution are dispersed, the integration of the self-governance of communities of shared environmental interests should be strengthened. The role of self-governance should be given full play at the community level in rural environmental governance and the effectiveness of endogenous order should be improved, as this would provide a source of strength for ecological development [46].

### 4.2. Fostering Villagers’ Sense of Participation in Environmental Protection

Rousseau (1999) stated that “in addition to these three categories of law there is a fourth, which is the most important of all; it is not graven in marble or bronze, but in citizens’ hearts; in it lies the true constitution of the state” [47]. Public participation is an important means of incorporating the state’s actual constitution into statutory law. For rural environmental governance, the participation and the actions of farmers are equally essential. Villagers are the direct victims of pollution, the closest observers of environmental issues, and the direct beneficiaries of environmental management. From the perspective of the farmer as the subject of rural environmental rights, they can be conceived as what Hart calls “a small-scale sovereign” with regard to the rights holder in the area of behavior covered by an obligation [48]. Only once villagers are organized to participate in the systemic project of rural environmental governance will they be able to share the responsibilities and benefits of rural environmental governance and finally reach a scenario in which all parties gain. Recognition of environmental protection and government policies and support are the prerequisites for villagers to participate in rural environmental governance. It is only once villagers’ heartfelt support and recognition have been gained that their enthusiasm can be stimulated and their ability to participate in environmental governance enhanced; hence, it is necessary to implement plans concerning environmental pollution and environmental governance in rural areas and set and achieve goals for rural environmental governance [49]. The subjective aspect of farmers’ participation in environmental governance needs to be brought into play at different levels [50].

In terms of the ways of gaining villagers’ recognition of environmental governance decisions, villagers and officials can conduct collaborative consultations on planning projects and regenerate the traditional Chinese spirit of mutual help and dedication. In addition, propaganda should be tailored to the environmental protection needs of villagers [19]. Cooperation and coordination will guarantee villagers’ participation in environmental governance. Environmental governance in rural areas involves consultation and co-governance among multiple stakeholders, which manifests in two ways. One is the fostering of an atmosphere of consultation among villagers and the promotion of their awareness of and ability to engage in environmental governance. The second manifestation is village–enterprise cooperation and consultation. Pollutant discharge by enterprises in villages is one of the most important contributors to environmental pollution in rural areas. In the process of treating pollution, villages can negotiate with enterprises on ways to discharge and the treatment of pollution to create a healthy living environment. Additionally, consultation between villages and governments should be upheld. In the project of environmental management provided by the government, the specific project implementation method should be changed from that of the previous government to complete the leading approach, establishing a consultation process involving villages and the government.

### 4.3. Balancing Economic and Environmental Interests in Rural Environmental Governance

Environmental governance involves a process of benefit gambling involving the economy and the environment. Since the beginning of time, humans have had a symbiotic, interdependent relationship with nature. However, in regions and times where productivity was relatively low, humans exploited natural resources for survival and societal progress, leading to environmental pollution and ecological imbalance. Consequently, an unhealthy relationship was formed between human development and natural protection, with natural disasters frequently occurring in response to human society’s excessive expectations. Around the world, the process of industrialization has also raised environmental awareness among different groups. The British government ultimately passed the Clean Air Act in 1956 as a direct response to the Great Smog of London in 1952, while the United States withdrew from the Paris Agreement, which addresses global climate change, on the grounds that it had harmed U.S. economic interests. In rural China, while environmental degradation is of increasing concern to Chinese policy makers, environmental goals are often seen as secondary to those related to food production and economic growth. However, the health costs of environmental damage should not be overlooked in policy design targeting the improvement of rural livelihoods [16]. In short, environmental governance must strike a balance between environmental interests and economic interests: “While each right advances the interests of the rights holder, yet the reasons for protecting those interests, and those evidentiary reasons for protecting the right, are not limited to concerns for the well-being of the rights holder” [51].

China’s rural environmental governance necessitates the formation of a community of shared environmental interests and must bring both environmental benefits and economic benefits into play. The establishment of a community of shared interests must take into account the common interests of the members in the region. It is impractical to force farmers to sacrifice economic benefits to protect agricultural ecology. This has nothing to do with their morals, nor is it just a matter of their environmental awareness; rather, at this particular social development stage, farmers’ needs for economic benefits far outweigh those for environmental benefits, which is the logic of survival. It was only after the national poverty alleviation policy was put into practice and the economic income of people in rural areas increased significantly that the demand for environmental benefits gradually became an immediate one.

Some provinces in China have already followed multi-actor and multi-layer patterns in environmental governance that have yielded fruitful results. For instance, in order to boost rural revitalization, Zhejiang province has accelerated the comprehensive improvement of the rural environment and promoted green growth supported by enterprises. Jiangsu province has adopted the urban–rural integrated ecological and environmental governance model, while other provinces have developed relocation models and enterprise engagement in environmental governance. The core of these successful governance models lies in balancing the relationship between farmers’ economic interests and environmental interests. Transferring land rights in a rural collective unit can vigorously improve coordination, increase agricultural efficiency, and ensure that chemical fertilizers and pesticides are employed on a sound basis, hence increasing farmers’ income. With the establishment of specialized farmer cooperatives, unified standards and sales and planned selling have been dictated, which ensure the market is provided with safe and guaranteed agricultural products. Involving farmers in specialized farmer collectives can also resolve the conflict between farmers’ employment and product standardization and provide increased income for farmers, food security, and land security. To sum up, personal interests and collective interests, as well as economic interests and environmental interests, form a unity of opposites. Conflicts of interests cause the primary contradiction in rural environmental governance and must be thoroughly considered and addressed.

### 4.4. Developing a Law-Based Rural Environmental Governance System

To establish a community of shared environmental interests with authority and effectiveness, law-based governance must be exercised, environmental laws must be introduced and strictly enforced, and justice must be administered impartially.

Rule of law is the pathway to achieving effective rural environmental governance. The environmental legal system provides guidance and interpretation for the goals, directions, and tasks of environmental legal governance; regulates the specific methods and rules of behaviors employed by environmental legal governance in rural areas; and determines and guarantees the rights and interests of related bodies under the rural environmental system [52]. Even a legal system with a substantial share of technical norms, such as the environmental legal system, is inherently part of the quest for fairness and justice in modern society [53]. In light of the conflict between unbalanced and inadequate legislative development, on the one hand, and the ever-growing need for legislation in rural areas and the gap in special legislation, on the other hand, the state has the responsibilities of strengthening rural environmental governance in terms of interest orientation and allocation of legislative resources and formulating comprehensive and targeted special legislation on rural environmental issues that is responsive to farmers’ real needs for environmental governance, as “responsiveness is the justification for democracy’s own legitimacy” [54]. Moreover, the government also has the responsibility of mobilizing social resources to promote the formation of a governance system led by the CPC committees that is implemented by the government based on consultation, broad participation, and the rule of law and supported by technology [55]. To move from a government-centered environmental governance mode to cooperation-based governance shared among multiple stakeholders, the leadership, promotion, and oversight of grassroots governments are indispensable.

In addition, regarding the judiciary, it is necessary to improve the level of professionalism among the ranks of judicial personnel in rural areas. To be able to handle complex environmental disputes, professional judges should be trained who are proficient in environmental resources protection. High-quality composite judicial teams can facilitate the efficient and appropriate resolution of rural environmental cases. As statutory environmental public interest litigation agencies, procuratorial organs also need to pay more attention to the current situation of rural environmental pollution, take the initiative to track down rural environmental pollution cases, and implement multiple measures to encourage farmers to actively submit disputes involving environmental protection to the procuratorate so that they can grasp the dynamics of rural environmental governance in a timely manner.

Finally, it is possible to construct new judicial mechanisms for rural environmental governance. For example, People’s Mediation Committees deal with relatively simple environmental disputes and help the parties reach an agreement on the basis of mutual understanding. In terms of operation, People’s Mediation Committees are guided by the Justice Bureau at the primary level. In any case, the arbitration tribunal for environmental disputes is mainly targeted at relatively complex environmental disputes. Compared to People’s Mediation Committees, the members of the tribunal are more professional; compared to judicial trials, the tribunal is more flexible, as the process involves both mediation and adjudication on the basis of the voluntary and consensual agreement of the parties.

## 5. Conclusions

Rural environmental governance is an important part of national governance and a problem in the rural revitalization strategy that must be solved. Rural environmental governance faces multiple obstacles, such as development model defects, the reality of funding shortages, and lagging governance concepts. Therefore, the key to rural environmental governance is to change the concept of governance and seek new ideas for rural environmental governance in terms of the concept of the community with a shared future. Rural environmental governance from the perspective of the community with a shared future demonstrates a new attitude. It is necessary to comprehensively solve the problem of rural environmental governance at the multiple levels of rural environmental governance concepts, governance subjects, legislative orientation, and judicial security. 

## Data Availability

Not applicable.
